# Surgical Morbidity in Relation to the Surgical Approach for Olfactory Groove Meningiomas—A Pooled Analysis of 1016 Patients and Proposal of a New Reporting System

**DOI:** 10.3390/brainsci13060896

**Published:** 2023-06-01

**Authors:** Ekkehard M. Kasper, Farhan A. Mirza, Serdar Kaya, Robert Walker, Daniele Starnoni, Roy T. Daniel, Ramesh Nair, Fred C. Lam

**Affiliations:** 1 Department of Neurosurgery, St. Elizabeth’s Medical Center, Brighton, MA 02135, USA; serdar.kaya@steward.org (S.K.); robert.walker@steward.org (R.W.); fred.lam@steward.org (F.C.L.); 2 Dana Farber Cancer Institute, Boston, MA 02215, USA; 3 Boston University School of Medicine, Boston, MA 02118, USA; 4 Division of Neurosurgery, Hamilton General Hospital, McMaster University Faculty of Health Sciences, Hamilton, ON L8L 2X2, Canada; 5 Department of Neurosurgery, University of Kentucky, Lexington, KY 40536, USA; fahan.amirza@gmail.com; 6 Neurosurgery Service, Centre Hospitalier Universitaire Vaudois/University of Lausanne, CH-1011 Lausanne, Switzerland; daniele.starnoni@unil.ch (D.S.); roy.daniel@chuv.ch (R.T.D.); 7 Neurosurgery Service, Charing Cross Hospital, Imperial College London, London W6 8RF, UK; rbrnair@gmail.com; 8 Harvey Cushing Neuro-Oncology Laboratories, Brigham and Women’s Hospital, Harvard Medical School, Boston, MA 02115, USA

**Keywords:** olfactory groove meningioma, outcomes, surgical resection

## Abstract

*Background*: There is currently no consensus in the field regarding whether a frontal or lateral approach is superior for microsurgical resection of olfactory groove meningiomas (OGM). Due to the lack of uniformity in classifying lesions and inherent differences in reporting outcomes after varying operative approaches, the best practice for approaching these lesions is yet to be determined. *Objective*: This study aimed to assess various surgical approaches undertaken for OGMs, investigate procedural aspects influencing the extent of resection, and analyze the respective complication rate associated with each approach. We performed a comprehensive literature review of presenting signs and symptoms in OGM patients, their surgical management, and the reported surgical outcomes. To address the lack of uniform data reporting across studies and to take more recent translational studies into account, we developed a new classification system for OGMs that can remedy the existing deficiencies in comparability of reporting. *Methods*: We conducted a PRISMA-guided literature search for surgical reports on OGMs published in the MRI era using broad search terms such as ‘olfactory groove meningioma’ and ‘surgery’, which yielded 20,672 results. After title screening and removal of duplicates, we assessed 871 studies on the specific surgical management of olfactory groove meningiomas. Following the application of exclusion criteria and abstract screening, a set of 27 studies was chosen for the final analysis of a pooled cohort of these reported patient outcomes. *Results*: The final twenty-seven studies included in our in-depth analysis identified a total of 1016 individual patients who underwent open microsurgical resection of OGMs. The approaches used included: pterional/unilateral, bifrontal with variations, and anterior interhemispheric approaches. Across all studies, gross total resection (Simpson Grades I or II) was achieved in 91.4% of cases, and subtotal resection (Grades III and IV) was reported in 8.6% of cases. A cumulative twenty-seven percent of surgical OGM patients sustained some form of complications. Minor issues accounted for 22.2% (CSF leak, seizures, infection, transient cranial nerve palsies, hydrocephalus), whereas major issues comprised 4.7% (hemorrhage, ischemic infarct, malignant cerebral edema). We then examined the correlation between these complications and the surgical approach chosen. Among pooled cohort of 426 patients who underwent unilateral approaches, 14% experienced minor complications, and 2.1% experienced major complications. For the mixed cohort of 410 patients who underwent bifrontal approaches, 24.6% experienced minor complications, and 7% experienced major complications. *Conclusions*: Unilateral approaches appear to have lower complication rates for the resection of OGMs compared to bilateral approaches. However, the extent of resection is not uniformly reported, making it difficult to identify differences. The use of an improved preoperative classification and scoring system can help establish a more coherent system to select the most suitable approach and to uniformly report surgical outcomes, such as EOR and complication rates specific to a given OGM and its surgical approach.

## 1. Introduction

Olfactory groove meningiomas (OGMs) constitute approximately 6–10%, and in certain series, as much as 18%, of intracranial meningiomas in tertiary brain tumor practices [1,2]. Since the first published account of a surgically removed OGM *intra vitam* by Francesco Durante in 1885, there has been an ongoing debate regarding the best surgical approach and management for OGMs of various sizes [3]. In the pre-imaging era, these tumors would often reach a massive size before being discovered. This changed with the introduction of early imaging via pneumo-ventriculography by Walter Dandy [4]. In 1935, Olivecrona in Sweden and Toennis in Germany subsequently described their experiences with a unilateral frontal approach to these tumors [5,6]. In 1938, Cushing and Eisenhardt described a similar technique in the USA, that involved partial resection of the basal inferior frontal lobe for better tumor exposure [7]. On the other hand, Dandy developed a more radical approach, performing a bifrontal craniotomy and a bilateral partial frontal lobectomy [8]. In subsequent years, several prominent figures in the field published their personal series illustrating varying approaches to this challenging tumor type [9,10,11,12]. However, at present, there is no apparent consensus in the field on which open approach is most suitable for the majority of OGM cases or which surgical strategy provides the best post-operative functional outcome. Due to the relatively infrequent occurrence of this particular meningioma type in most centers, large-scale studies to determine the best surgical approach have not been conducted.

Another important observation made during this study is the significant variation in reporting among published series regarding pre-operative symptoms and the radiographic modalities used for the description of these lesions and their respective features. This leads to the utilization of a variety of categorizations by size, osseus involvement, extent of resection, and respective procedural complications. Consequently, this variation impacts the reporting of post-operative outcomes and the assessment and definition of recurrence rates. Furthermore, most of these details and measures are not reported separately for each approach chosen in many of the available series, which creates a major limitation to conducting a truly systematic review and/or meta-analysis on the topic. Although this has previously been attempted in a general sense by authors grouping various approaches into either unilateral or bilateral approaches [13,14], we believe that such grouping blurs the distinct strategic advantages provided by each microsurgical technique. Therefore, we undertook this review to address these limitations.

With these limitations in mind, we decided to proceed with an in-depth and comprehensive review of relevant publications on the topic. We collected a pivotal data set from seminal existing series which met specific inclusion criteria. We then applied a cross-sectional analysis technique to bundle individually reported OGM patients based on the approach used. This approach allowed us to perform a subsequent subgroup analysis to derive clinically meaningful results specific to each of the approaches reported.

To address the underlying problem of lesional variations, we endeavored to provide a new classification system and checklist which could be applied to the clinical and radiographic assessment of OGM patients preoperatively. This initiative aims to make future clinical cohort studies comparable to surgical outcomes in a meaningful way.

## 2. Methods

### 2.1. Data Sources

**Figure 1:**
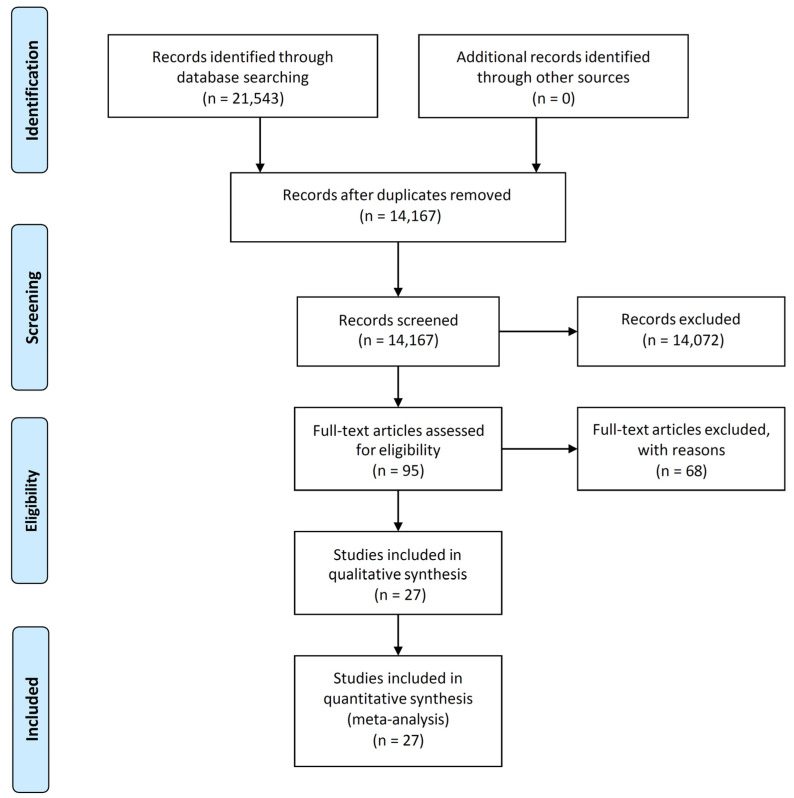
PRISMA flow diagram.

A PRISMA-guided literature search for the MRI era (defined as the time after 1990, when MR-Imaging was widely introduced into clinical practice) was conducted in MEDLINE (PubMed) and EMBASE, with the following search terms: ‘olfactory groove meningioma’, ‘surgical management of olfactory groove meningioma’, ‘intracranial meningioma’ and other variations applied in a Boolean fashion. Additional searches were conducted in Google Scholar to avoid missing articles that lacked one of the search terms in the title, abstract, or index terms. Finally, an extensive manual search was performed starting with the references of all the included studies and relevant reviews on this topic, which were then screened for eligible studies. Please see Figure 1 below.

### 2.2. Eligibility Criteria

Eligible studies had to meet the following criteria to be included for review: (1) published between January 1990 and March 2018, supplemented by a re-run update in December 2022, (2) studies had to be on human subjects, (3) written in the English language, (4) including more than five adult patients, and (5) manuscripts had to be classified as full-text, peer-reviewed papers in indexed journals. No restriction was applied regarding the design of the study. Publications written in languages other than English were excluded for this pilot project. F.A.M. and E.M.K. screened all the retrieved titles and abstracts. Differences in assessment for analysis were resolved with assistance from a third assessor (F.C.L.). A final selection of articles for granular analysis was then made by both F.A.M. and E.M.K. based on the review of full-text papers.

### 2.3. Data Extraction

The data extraction was performed by F.A.M. and E.M.K. The following information was extracted both on a study level and on an individual patient level: (1) the reported surgical approach (if bilateral, then also whether sub-frontal, interhemispheric, or bifrontal; if unilateral, then also whether pterional, frontolateral, subfrontal, or supraorbital = eyebrow approach); (2) pre- and postoperative symptoms and the extent of resolution/improvement therein (olfaction, cognitive changes, headaches, seizures, visual changes); (3) postoperative complications; (4) pre- and postoperative reports of imaging findings (specifically from CT and MRI-FLAIR images); and (5) intra-operative brain relaxation strategies (lumbar drain, lumbar puncture, mannitol, furosemide, hyperventilation). The extracted data were recorded in pre-developed tables in MS Excel and Word.

## 3. Results

The broad search term ‘olfactory groove meningioma’ yielded 20,672 initial results on PubMed in 2018, whereas ‘surgical management and olfactory groove meningioma’ revealed 871 results across all queried data bases. After removing duplicates and unrelated studies, 14,167 titles were reviewed, and 95 studies were selected for abstract and content review. Of these, 35 papers were further selected for full text review. The remaining 60 papers were excluded, as they addressed exclusively endoscopic techniques (eleven manuscripts), reported only on single cases (thirty-nine), were published in another language (five), or full-text access and author contact information was not available (five). Of the 35 extracted full-text papers, only 27 met all the inclusion criteria to proceed with analysis. We also did not include studies in our pooled cohort that described patients undergoing both combined open and endoscopic surgical procedures, as we found a significant selection bias with each technique. An extensive review by the EANS skull base committee conducted in 2022 [13] has addressed this topic, and we refer the interested reader to that particular study for further comparison. For ease of reference, we have summarized the 27 papers used in the pooled analysis in Table 1.

In a recent 2022 re-run of our search, no significant new results from interim publications were identified which would have altered the results or conclusions of our initial analysis. Given the complexity of the analysis, we decided not to reperform the statistics. However, for completeness, we included several further cohort and review studies from the updated search in the introduction and discussion sections of the current work.

Lastly, due to the heterogeneity of the data presentation found in almost all of the studies selected for final analysis, a uniform statistical analysis was not possible, and a true meta-analysis could not be performed. For this reason, to achieve a meaningful, comprehensive analysis and interpretation, we proceeded to pool the data from individual studies based on approach type, which allowed us to obtain frequencies and percentages of different variables of interest. We also grouped studies which described one particular OGM resection approach exclusively for all their included patients to better analyze the results affiliated with each approach. In cases where this was not possible, we made comparisons by grouping different approaches into ‘bilateral’ (bifrontal, subfrontal, etc.) and ‘unilateral’ (pterional, lateral, etc.). In the final set of 27 selected studies published after 1990, a cumulative total of 1016 patients undergoing microsurgical resection of OGM of varying dimensions (ranging from 1.5–10 cm) were reported in detail.

### 3.1. Presenting Symptoms

The lack of uniformity in preoperative symptom reporting in this pooled study cohort prevented us from analyzing the true incidence of all cardinal symptoms (headache, olfaction, cognitive symptoms, seizures, visual symptoms) separately for all of the 1016 cases. Some studies did not report symptoms for each of the patients included in the final manuscript. Therefore, we grouped the reported individuals from all studies in which each symptom was recorded separately and combined these study cases to determine at the relative cumulative incidence of each symptom.

This analysis yielded the following reported % frequencies for the five cardinal presenting symptoms: altered olfaction: 59.6% (526/882), visual changes: 50.4% (447/886), cognitive changes: 47.8% (415/868), headache: 43.2% (338/781), and seizures: 17.4% (134/767).

### 3.2. Approaches Reported

The following approaches were utilized for the 1016 pooled cohort patients: bifrontal (456 cases, 44.88%); pterional (308 cases, 30.30%); unilateral-frontal (66 cases, 6.49%); and variations of fronto-orbito-basal, subcranial, or transfrontal/transsinus (75 cases, 7.38%). A separate group was established for anterior interhemispheric cases (18 cases, 1.77%). A total of 9.18% of cases remained poorly specified.

### 3.3. Extent of Resection

Gross total resection, as described in most studies according to Simpson Grades I or II, was achieved in 929/1016 of the reported cases (91.4%), whereas subtotal resection (Simpson Grades III and IV) was achieved in 87/1016 cases (8.6%). Of note, the extent of the bony removal was not adequately described in detail in most of these studies. With this limitation in mind, the overall recurrence rate over the (variable) follow-up period was 4.9% (reported in 50/1016 cases). However, it should be acknowledged that data loss is likely, and the available data may not accurately reflect approach-specific outcomes.

To address this issue, we examined a smaller subset of studies reporting a single approach for all their patients or provided separate recurrence rates for each employed approach. The following gross total resection rates were noted:

1. Interhemispheric (18/18 cases, **100%**) [16]
2. Pterional (6/6 cases [17], 20/20 cases [18], 37/37 cases [19], 17/18 cases [30], 60/61 cases [34], for a total of 140/142 cases, **98.5%**)
3. Bifrontal (11/13 cases [21], 14/15 cases [22], 17/17 cases [25], 31/35 cases [27], for a total of 73/80 cases, **91.2%**)
4. Frontolateral (14/18 cases [29], 60/66 cases [9], for a total of 74/84 cases, **88.1%**)
5. Subcranial variations (21/21 cases [39], 12/19 cases [31], 22/2 cases [36], for a total of 55/62 cases, **88.7%**).

### 3.4. Complications

Across all studies contributing to our pooled cohort of 1016 patients, a substantial number, 274/1016 patients (27%), sustained some form of complication. Dividing these into major/life threatening complications and minor/non-life threatening complications, 48 cases (4.7%) were complicated by major issues (defined as hemorrhage, ischemic infarct, venous infarct, or malignant cerebral edema), and 226 (22.2%) patients sustained minor issues (defined as CSF rhinorrhea, seizures, infection, cerebral edema managed conservatively, pneumocephalus, transient cranial nerve palsies, hydrocephalus requiring external ventricular drainage, or shunting). Further details include:

1. Major/life-threatening postsurgical hemorrhage occurred in 32/1016 patients in our cohort (3.1%). A debilitating ischemic infarct occurred in nine patients (0.88%), and malignant cerebral edema occurred in ten patients (1%). Mortality attributed to direct neurological causes was identified in 12 patients (1.18%). Overall mortality, including deaths from non-neurological causes in the peri-operative period, was reported for 22 patients (2.2%).
2. Minor complications reported in the full pooled cohort included: 60 cases of CSF leaks (5.9%); 29 cases of infection, meningitis and/or osteomyelitis (2.85%); 49 cases of post-operative seizures (4.8%); and 23 cases of hydrocephalus (2.26%) requiring temporary external ventricular drainage or permanent CSF diversion. Non-life-threatening cerebral edema/brain contusion occurred in 30 cases (2.95%). Post-operative cranial nerve deficits, such as transient palsies and visual deficits, developed in 15 patients (1.47%).
3. We excluded anosmia in this listing due to inconsistencies in reporting.

To provide a more clinically meaningful perspective, we then examined specific complications associated with each particular approach:

1. Pterional approaches: Eleven of 13 studies (308 patients total) reported distinct complications, for a total of 298 patients (96.8%) [15,17,18,19,24,26,27,30,32,34,36,37,38]. Of these, minor complications were noted in 25 patients (8.3%), including anterior pituitary insufficiency (one case), infection (four cases), seizures (six cases), hydrocephalus (five cases), CSF leak (two cases), and brain contusion (two cases). Major complications occurred in five patients (1.6%), including: ischemic infarct (two cases) and hemorrhage (three cases). No mortality was noted using the pterional approach.
2. Frontal approaches: Seventeen studies utilized a bifrontal (456 cases, 44.88%) or unilateral-frontal (66 cases, 6.49%) approach [1,20,21,22,24,25,26,27,28,31,32,33,35,36,37,38,39]. In the bifrontal group, four studies (74 cases) used a subcranial approach: Spektor (12 subcranial) [23], Pallini (22 orbitofrontobasal) [36], Pepper (19 subcranial) [31], and Barzaghi (21 trans-frontal sinus subcranial) [39]. Thirteen studies reported detailed complications with either bifrontal or non-descript “unifrontal” approaches in 410 patients. Of these, 101 patients had minor complications (24.6%), including cranial nerve palsies or deficits (ten cases), infection (17 cases), seizures (14 cases), hydrocephalus (nine cases), CSF leak (26 cases), cerebral edema (22 cases), and pneumocephalus (three cases). Major complications occurred in 29 patients (7%), including: ischemic infarct (four cases), hemorrhage (21 cases), and malignant cerebral edema (four cases). Ten mortalities (2.3%) from direct neurological causes were noted with this set of approaches. A separate analysis was done for the anterior interhemispheric approach, since only one substantial study, containing 18 patients, has been published using this approach at the time of the manuscript preparation [16]. One minor complication (infection), and one major complication (ischemic infarct of the medulla leading to coma and death four months later) occurred in this series (5.5%).
3. Supraorbital or frontolateral approaches: Seven studies described the use of a frontolateral/supraorbital approach in a total of 168 patients (16.5%) [9,24,26,28,29,36,38]. Four studies exclusively reported their complications with this approach in 128 patients (76.2%). Minor complications occurred in 35 patients (27%), including subdural hygroma (six cases), hydrocephalus (four cases), cerebral edema (one case), seizures (four cases), CSF leak (11 cases), visual deficits (five cases), and infections (four cases). Major complications occurred in four patients (3.1%), including hemorrhage (three cases) and one case of ischemic infarct secondary to anterior cerebral artery (ACA) injury resulting in death (0.78%).
4. Fronto-orbito-basal approach: 22 craniotomies out of 113 in one series were performed using this approach [36]. Minor complications were noted in ten (45.4%) patients (CSF Leak (4), wound infection (1), seizures (1), cerebral edema (4), hydrocephalus (1), and one major complication (hemorrhage)). No mortality occurred.
5. Trans-frontal sinus subcranial approach (TFSSA): One hemorrhage was noted in this series of 21 patients [39]. No minor complications or mortality were noted. For ease of reference, we have summarized the post-operative complications reported for each approach in the 27 studies in Table 2.

## 4. Discussion

Olfactory groove meningioma resection remains a challenge, even in the hands of an experienced surgical team. It is well established that good outcomes are accomplished by surgeons with high-volume practice who have extensive hands-on experience with meningiomas. The principles of resection of OGM resection revolve around the same aspects that apply to other neoplastic lesions involving the anterior skull base: early identification and diligent coagulation of the blood supply to the tumor, early identification of neurovascular structures, dissection and atraumatic handling of the frontal lobes, and meticulous preservation of venous drainage.

An ideal surgical approach for OGMs, regardless of size, should facilitate minimal brain retraction, preserve normal tissue and integrity of neurovascular structures, and allow reconstruction of the skull base as needed [40]. However, several factors need to be taken into account when choosing a particular approach for these tumors.

### 4.1. Presenting Symptoms and Preoperative Radiographic Appearance

Zygourakis et al. conducted a study on their mixed series of 44 olfactory groove and planum sphenoidale meningiomas in order to identify clinical and imaging factors pertaining to the tumor that may predict the persistence of symptoms and/or post-operative complications [41]. The most common symptoms in their series included visual problems (36%), headaches (30%), personality changes (21%), seizures (11%), and anosmia (9%). In the largest relevant series on the topic to date by Pallini and colleagues, the most frequent complaint was anosmia (59.6%), followed by visual impairment (46.5%), headache (38.4%), mental changes (35.4%), and seizures (19.2%) [36].

These findings align well with our pooled analysis of 1016 patients, where the following distinct symptoms were noted in decreasing order of frequency: decreased sense of olfaction (59.6%), visual changes (50.4%), cognitive changes (47.8%), headaches (43.2%), and seizures (17.4%).

Preoperative assessment of olfactory function is both vital in surgical decision making and when assessing and reporting outcomes (Dedeciusova et al., 2020) [14]. Preoperative normal olfactory function seems to be the most relevant factor in functional postoperative outcomes. Higher tumor volumes were found to be associated with the size of the lesion. Anatomical preservation of the structural integrity on the side contralateral to the surgical approach appeared to be the key feature in the preservation of the sense of smell. This has been previously noted by Welge-Luessen et al., who reported in their series that post-operative perseveration of contralateral olfactory function was related to the pre-operative status and size of the tumor (<3 cm) [20]. They also observed that it is difficult to anatomically preserve the olfactory tract ipsilateral to the tumor during resection, but even if preserved, functional restoration was unlikely. Jang et al. noted that a tumor size of <4 cm correlated with olfactory function preservation in their series (reporting preservation in 78.6% of patients with tumor sizes < 4 cm and 42.3% in sizes > 4 cm) [33]. It was also noted that olfactory nerve sparing was significantly higher in patients without preoperative olfactory dysfunction (84.6%) compared to those with pre-existing dysfunction (40.7%, *p* = 0.016). In this series, the frontolateral approach achieved better post-operative olfactory function (71.4%) than the bifrontal approach (36.8%) [33]. However convincing, these findings were not corroborated by an extensive systematic review and meta-analysis. De Oca and colleagues could not demonstrate this correlation due to the lack of detail in reporting. The authors suggested further investigation of this issue with the introduction of an experimental control [14].

Cognition, mental status changes, and visual problems usually improve significantly after the removal of these tumors. Gazzeri et al. utilized the Mini Mental Status Examination (MMSE) as a pre- and post-operative evaluation tool to quantify cognitive status as well as to assess the impact of a bifrontal approach [27]. The vast majority of patients showed significant recovery of mental function to near normal values after surgery [27]. This was confirmed in a more recent 2018 study by Fountas and colleagues, who reported their experience with a large, pooled cohort (*n* = 78) from two European centers. The patients’ mental status was assessed via MMSE and a second instrument, the frontal assessment battery (FAB). Although the MMSE scores improved significantly in this study, the FAB scores did not change significantly, though a correlation approach was not used. Furthermore, detailed neuropsychological outcomes were reported by Constanthin and colleagues in their 2021 paper which studied a small mixed cohort of 17 OGM patients [42]. Unfortunately, no surgical details were provided. In this report, the authors found that presurgical cognitive impairment was observed in most patients, particularly within the domain of cognitive flexibility. Despite clear heterogeneity between individual patient scores, all patients showed an immediate worsening of cognitive function postoperatively across all the tested domains, most significantly in measures of attention and cognitive flexibility. Despite general improvements over time, the 12-month follow-up assessment revealed ongoing impairment in attention and flexibility scores. It remains unclear whether these results correlate with any imaging sequelae postoperatively, such persistent bifrontal T2/FLAIR abnormalities on MRI.

Postoperative visual worsening has also been reported in several series. One noteworthy case was described by Hentschel et al., where a patient experienced delayed deterioration of vision [21]. This patient was treated with blood pressure elevation and calcium channel blockers, as it was believed to be caused by vasospasm of perforators to the optic nerves. The patient’s symptoms completely improved after treatment, corroborating this concept. The overall reported visual improvement rate in the study by Gazzerri et al. was 25.6% for acuity and 28.5% for visual field defects. Zygourakis et al. noted that patients with tumors close to the optic chiasm (defined as reaching <6 mm from the tumor edge to the optic chiasm) were more likely to have visual symptoms than those with tumors further (>6 mm) from the optic chiasm [41]. The authors did not comment on any impact this might have on surgical strategy or management.

### 4.2. Resection Strategy

Likely the most important consideration in deciding on a particular surgical approach is the surgeon’s personal experience and his/her familiarity with not only the technical aspects of approach, but also the anatomy of the lesion and areas involved, as seen from the particular chosen angle. The mastery of technical nuances of each approach and the surgeon’s expertise has an obvious impact on achieving superior outcomes. This was briefly mentioned by Pallini and colleagues in their analysis of surgical experiences and post-operative outcomes, where they noted a correlation between lower levels of life-threatening complications and more extensive experience of the respective surgeons [36]. We would like to elaborate on this by summarizing patient details and then providing comments on certain surgical concepts and approaches below.

### 4.3. Intraoperative Brain Relaxation Strategies

In their series of 28 patients from 1994, Schaller et al. employed a pterional approach in all cases, as originally advocated for by Hassler and Zentner [10,15]. In all operations, these authors placed a lumbar drain for brain relaxation. Similarly, in their description of their bifrontal technique, Colli et al. mentioned the use of routine lumbar drainage [25]. As evidenced in our comparison, the rate of subsequent CSF leaks is much lower with the pterional approach when compared to the other frontal approaches. However, a lumbar drain may be very useful not only for intra-operative relaxation, but also to prevent or address postoperative CSF leaks. It is commonly used after vascular or endoscopic skull base surgery, where drilling of the sella or anterior skull base occurs, and a ‘gasket seal’ or multilayered tissue closure is performed to prevent post-operative CSF leaks [40]. In the majority of large OGM cases, the anterior skull base needs to be drilled extensively to achieve a complete resection. In this setting, the probability of a CSF leak increases significantly with the use of a bifrontal technique, as seen in our analysis. Thus, the use of a lumbar drain may be helpful in reducing the incidence of this complication from the onset. The brain relaxation that can be achieved with this adjunct should also significantly impact the rate of brain contusions and retraction-related cerebral edema. Furthermore, the injection of tracers (such as fluorescein) can significantly help in identifying CSF leakage intraoperatively as well as postoperatively.

### 4.4. Unilateral Approaches

#### 4.4.1. Pterional Approach

The pterional approach was pioneered by Yasargil in 1967 [43]. Based on their observation of Seeger’s diligent skills, two of his followers, Hassler and Zentner, reported the first relevant series of OGMs resected using this technique in 1989 and referred in their paper to its evolution from Seeger’s bilateral technique, which had been used preserving the midline [10]. The pterional approach allows for early identification of neurovascular structures, facilitating the preservation of ACA perforators while the tumor is systematically devascularized [15]. This is not equally feasible with all frontal approaches. The frontal sinus and large draining veins are avoided in the pterional approach, and frontal lobe retraction is minimized. CSF spaces are entered early on, which allows for ample brain relaxation. Tumor extension into the ethmoid sinus is somewhat more challenging, but can occur in a large number of cases [5,9,21,23,26,29,31,32]. In these cases, a more recent combined lateral and endoscopic endonasal resection of the ethmoidal extension may be beneficial in minimizing complications otherwise associated with more extensive subfrontal approaches. However, an extended fronto-temporal craniotomy and orbital osteotomy, as proposed by Sekhar and colleagues (also known as the *one and a half* approach), may be utilized in this instance to obtain a more expanded view of the anterior skull base [24].

In d’Avella’s study from 1999, six cases of massive (>6 cm) OGMs were operated upon using a pterional transsylvian approach [17]. Excellent outcomes were noted in all these patients. Anatomical preservation of the contralateral olfactory nerve was also achieved in five of the six cases. No patients suffered worsening vision, and pre-existing visual deficits improved in all patients. Although this comprised only a very small series, it highlighted the use of a unilateral approach for massive lesions with excellent functional outcomes. In this study, the frontal lobe changes noted on post-operative T2 and FLAIR MR imaging were also highlighted for the first time in the English literature. In 2011, the same group presented a follow-up analysis in which they quantitatively analyzed the volumes of post-operative porencephalic cavities and the efficacy of the pterional approach in minimizing frontal lobe damage compared to frontal approaches, even for massive lesions [30]. Similarly, in 1999, Paterniti et al. published their results on twenty OGMs [18]. These authors again emphasized outstanding results, describing Simpson Grade I resections achieved in all cases, no reported neurological complications, and no recurrence. In the same year, Turazzi et al. published their results on thirty-seven patients [19]. Again, no significant neurological complications were noted, and no recurrences were seen on long-term follow-ups. They also noted good preservation of the contralateral frontal lobe on post-operative imaging. In their series of both bifrontal and pterional approaches, Bassiouni et al. noted no major complications with the latter approach [24]. In another large series, Bitter et al. presented their experiences over two decades of using the pterional approach [34]. Again, these authors noted excellent gross total resection rates of 98%.

#### 4.4.2. Supraorbital/Frontolateral

The supraorbital approach was first described by Fedor Krause and elaborated upon by Perneczky and colleagues for a variety of skull base lesions [44,45]. This was further popularized by Hernesniemi and colleagues in Finland [9]. Good outcomes were noted in their series of 66 patients, all operated on using a lateral supraorbital approach. In their large series of 82 patients, Nakamura et al. described the use of the frontolateral approach in 34 patients and the bifrontal approach in 46 patients [26]. The authors clearly demonstrated a higher safety profile of the frontolateral approach, with no incurring major complication or mortality compared to the bifrontal approach. Comparable tumor sizes were noted for each approach. The mean diameter of their tumors was reported as 4.16 cm (range, 1.4–8 cm) in the frontolateral approach group and 4.89 cm (range, 2–10 cm) in the bifrontal group. Eleven tumors (32.4%) were smaller than 4 cm in the frontolateral group, and 11 tumors (23.9%) were smaller than 4 cm in the bifrontal group. Paranasal tumor involvement was present in two patients in the frontolateral group and in 14 patients in the bifrontal group. This inferior extension was likely the reason that the bifrontal approach was selected in these patients, allowing for maximal resection and reconstruction.

In 2009, El-Bahy described the use of the frontotemporal approach in his series of 18 patients, which were divided into two groups: Group A, with tumors less than 4 cm (7 cases); and Group B, with tumor size more than 4 cm (11 cases) [29]. Subtotal resection was performed in four patients due to encasement of the ACA in three cases and paranasal sinus extension in one patient. Jang et al. noted better olfactory preservation with the frontolateral approach compared with the bifrontal approach [33]. In their series, Banu et al. utilized three different techniques: endonasal, supraorbital eyebrow craniotomies (microscopic with endoscopic assistance), and combined endonasal endoscopic with either a bicoronal or eyebrow microscopic approach. Significantly less morbidity and recurrence were noted in the supraorbital craniotomy group [40].

### 4.5. Anterior Approaches

#### 4.5.1. Bifrontal Surgeries

Historically, the bifrontal craniotomy has long been the primary approach for anterior cranial base lesions. Several variations have been described in the literature [6,12,15,16,22,46]. Feiz-Erfan et al. proposed a classification system of transbasal skull base approaches in 2008 by dividing the several approaches that have been described into three levels (Level I, II, and III) [47]. Despite excellent tumor exposure, there are certain downsides to these approaches. The frontal sinus is almost always entered during the craniotomy, which will require elaborate repair if violated. The anterior superior sagittal sinus also requires ligation in most cases, which can contribute to significant postoperative bifrontal edema, as illustrated by Nakamura et al. [26]. The cisterns are also not reached early during dissection, though this effect can be countered with the use of lumbar drainage. The neurovascular structures located deep in the tumor are encountered at the end of the operation, which may lead to unintentional injury to these structures, as noted in two series [24,29].

In a study of 13 patients by Tsikoudas and colleagues, 11 were operated upon using a bifrontal technique, and unilateral-frontal technique was used in two patients. CSF leakage was the most common complication. One patient suffered blindness post-operatively. Four recurrences were noted on long-term follow-up (three, nine, ten, and eleven years) [1]. Similarly, in Bassiouni’s series, three CSF leaks were noted in the bifrontal craniotomy group [24]. Two patients suffered serious injury to the ACA, resulting in debilitating infarcts and disability. No CSF leaks or recurrences were noted in Hentschel’s series [21].

#### 4.5.2. Extended Subcranial Approaches

Pepper et al. presented their novel report of 19 cases in 2011 using a transglabellar subcranial approach [31]. The mean follow-up time in this series was 41 months. They noted a comparable extent of resection and complication rates, but noted significantly longer operative times, averaging 13 h. Additionally, five of their 19 cases required craniofacial reconstruction for contour defects or for post-surgical infections sustained from the initial approach [1,26,27]. In the current era of minimally invasive approaches with an increased focus on maximal safety and efficacy, this approach has been almost abandoned and considered unnecessary, as other less-invasive approaches appear to provide similar or better results. However, the approach remains useful in select patients whose tumors cannot be approached from above for anatomical reasons.

In 2013, Boari et al. published their cadaveric studies of a transfrontal sinus approach [48]. The same group presented their series of 21 patients undergoing a transfrontal sinus subcranial approach (TFSSA) in 2017 [39]. This approach was evaluated favorably, as it was associated with minimal morbidity, and no mortality was noted. Recent reports from the latest Pan African Association of Neurological Surgeons (PAANS) meeting presented by Gay and colleagues from Grenoble may lead to further resurrection of this technique (personal communication). In their large series, Pallini et al. utilized a pterional, bifrontal, or fronto-orbito-basal craniotomy. For tumors 3 to 6 cm and >6 cm in size, they noted higher gross total resection rates using a combined fronto-orbito-basal approach, allowing for no retraction-related frontal lobe injury or edema. However, this result yielded higher rates of CSF leakage [36]. The bifrontal approach had a higher morbidity rate when compared with the other two approaches in this series, but a higher number of patients required re-operations for various reasons in the fronto-orbito-basal group (22.7%).

#### 4.5.3. Interhemispheric Approaches

Only three papers have been published using this approach (see Montes de Oca, 2022) [13]. Of these, the only substantial English language case series on this technique was reported by Mayfrank et al., involving a unilateral paramedian approach [16]. The authors noted relatively short surgical times (average 230 min), early identification of dural feeders, good access for devascularization, no opening of the frontal sinuses, which eliminated the risk of any CSF leak, and good viewing angles, which facilitated a more efficient dissection of the neurovascular structures. The potential concerns with this approach include the chances of injury to the superior sagittal sinus, as well as frontal lobe retraction trauma. However, in their report, gross total resection was achieved in all patients, without any evidence of damage to the frontal lobes or neurovascular structures. This approach warrants further investigation after the application of a more coherent lesional classification system.

### 4.6. Transnasal Approaches

Although our current study primarily compares open surgical approaches to resection of OGMs, we must mention the ability to safely and effectively resect OGMs using the transnasal approach. We found three studies comparing open and transnasal approaches (Table 3). Based on these three studies, we conclude that the transnasal approach had a higher incidence of reported anosmia. This was recapitulated in a recent systematic review and meta-analysis of ten comparative studies, which concluded that transnasal approaches resulted in significantly less likelihood of worse vision when compared to transcranial approaches for OGM, with significantly higher incidences of loss of olfaction and CSF leakage [49].

### 4.7. Postoperative Complications in Relation to the Chosen Approach

In our pooled analysis of 410 patients undergoing a bifrontal approach in 13 distinct studies that reported complications separately for this approach, 101/410 patients were noted to sustain a minor complication (24.6%). Among these, CSF leak and cerebral edema requiring medical management were the most common problems encountered. In his series of 35 such patients, El Gindi noted that seizures were the most common postoperative complication [2]. In some series, CSF leaks were treated conservatively, either with serial lumbar punctures or the insertion of lumbar drains. Though no rate of persistent leakage was reported, it was mentioned in several cases that re-operation was required for definitive treatment [29,32,34,41,51,52].

Major complications occurred in 29 patients (7%) undergoing a bifrontal operation. Despite modern management options, a total of ten mortalities (2.4%) occurred, which could be traced to direct neurological causes. In El-Bahy’s series, four patients had significant ACA encasement, which led to subtotal resection in three patients. One patient suffered a debilitating infarct from ACA injury [29]. Zygourakis et al. noted that patients with ACA encasement were significantly more likely to experience postoperative complications compared to those without (50% vs. 7.9%) [41]. Similar findings were noted in patients who displayed tumors with paranasal sinus involvement, irrespective of tumor size or volume. Mukherjee et al. examined factors that could be predictive of post-operative complications [51]. In their analysis, the authors identified the presence of a ‘sabretooth’ radiographic feature of edema as a predictor of poor outcomes. This severe and striking perilesional edema was described as a peritumoral edema that extends through white matter tracts of the external capsule (between the claustrum and lentiform nucleus) towards structures of the diencephalon including the thalamus, thus depicting a characteristic triangular appearance similar to that of a sabretooth. Its preoperative radiographic presence was clearly associated with a higher risk of postoperative complications. It is interesting to note that small or medium-sized tumors (described as <4 cm in their series) with the presence of sabretooth edema were also associated with a higher perioperative risk compared to larger tumors displaying only mild to moderate edema [51]. Please refer to Table 2 for a summary of postoperative complications based on this approach.

## 5. Recurrence

In 1957, Simpson described a grading system for the resection of meningiomas [53]. In this seminal paper, it was noted that the risk of eventual recurrence after Simpson Grade I, II, III, and IV resections was 9%, 16%, 29%, and 39%, respectively, given a minimum of a six-month post-operative survival period. However, the relevance of this grading system has been questioned in the current era of modern neurosurgery, with enhanced operative tools, molecular markers, and stereotactic radiosurgery available to treat small recurrences [54,55]. In our mixed cohort study, we calculated a pooled recurrence rate of 4.9% (50/1016) over the reported follow-up range. However, this finding is questionable, as recurrences were not defined systematically, and follow-up duration was not described in all the studies. In the papers reviewed, insufficient information was given to analyze how the recurrences were managed. This will be the topic of a future study conducted by our group.

### 5.1. Influence of World Health Organization Grading on Recurrence Rate

As noted by Sughrue et al. in their experience with grade I meningiomas (not limited to the skull base), the reported modern recurrence rates were much lower compared to historical cohorts. The five-year recurrence or progression-free survival for all patients receiving Simpson Grades I, II, III, and IV resections was 95%, 85%, 88%, and 81%, respectively. No significant difference was noted in recurrence-free survival in all patients who had been followed for four or more years [55]. This differs from the findings of Oya et al., who reported that tumor control rates did not differ significantly, irrespective of tumor locations, whether Simpson grade I, II, or III resection was achieved, as long as gross total resection was achieved [54]. The only significant difference they reported was a shorter recurrence-free survival with grade IV resections or a subtotal resection. We agree with the assessment of both groups that maximal safe tumor resection, decompression of neurovascular structures, and minimizing patient morbidity is of greater importance than overly aggressive efforts to resect dura and bone at the skull base to achieve a higher Simpson grade at the cost of functional preservation. With more recent adjuvant therapy options with 3D conformal IMRT or SBRT, residual disease can be treated effectively, resulting in prolonged local control rates, which may be a favorable treatment strategy in the mostly elderly OGM patient population [56].

### 5.2. Limitations of This Study: Heterogeneity in Reporting of Tumor Size, Radiographic Characteristics, Details of Approaches, and Outcomes

In our analysis, the major limitation we faced was the heterogeneous and highly variable reporting of presenting symptoms, measures of tumor size, and tumor configuration. These include factors such as the extent of infiltration of surrounding structures, including soft tissue and bone, encasement of adjacent structures, edema pattern, resolution or worsening of clinical status post-operatively, post-operative imaging assessment, follow-up schemes/periods, recurrence rates, and details about particular approaches chosen.

The scenario is further complicated by the fact that, in many studies in which two or more surgical approaches were utilized, many authors did not group patients into subgroups consisting of those presenting symptoms or complications or by the operated approach. This made granular analysis difficult, and many reviews have failed to address this aspect. Only Montes de Oca and colleagues have attempted to address this question in some detail thus far [13].

### 5.3. Recommendation for Future Reporting and Proposal of New Classification System

Despite the sizeable number of studies available on this topic, to date, there remains no consensus in the field about the most suitable management of these formidable lesions. Currently, most studies have relied on a classification scheme of these tumors based on size range alone. We do not believe that this measure is clinically meaningful in isolation. These tumors are relatively uncommon, which makes it unlikely that large cohort studies will become available in the foreseeable future. Therefore, the best approach for this problem would be to create a consensus in the professional societies and to establish a uniform reporting method for case series of all anterior skull base meningiomas. This objective has recently been initiated by the EANS skull base committee (Michel Bruneau, personal communication).

**Figure 2:**
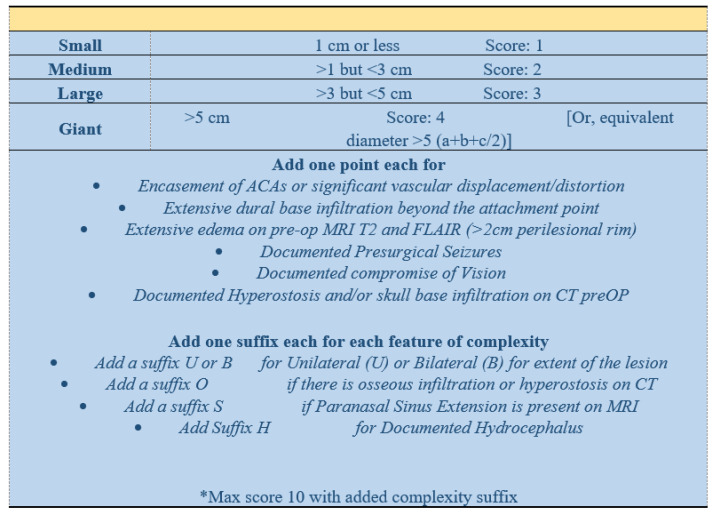
Proposed anatomical classification system for OGMs.

To address this significant variability in data reporting, we propose a new descriptive tool, which is outlined below and illustrated in Figure 2. This tool can guide neurosurgeons in selecting a suitable operative approach for OGM resection, as it is based on a selection process focused on minimizing surgical morbidity. An easy-to-follow checklist has also been developed to record patient data and to streamline data reporting in case series which can be provided upon request. In the future, we recommend consistent and systematic reporting of the factors described below.

All preoperative symptoms (with a clear statement of the status of olfaction, formal visual field, and acuity assessment, as well as a quantifiable mental status assessment) should be reassessed postoperatively using the same assessment instruments, along with pre- and postoperative Karnofsky Performance Status Score to assess functional outcomes and QOL.

Tumor size: Tumor size needs to be defined uniformly in all three radiographic planes for OGM and should be endorsed by the neurosurgical community. Presurgical MRI and CTA data, such as edema extent, pattern of calcification, hyperostosis, vascular supply, and encasement of critical structures, should be included in the description to complement the data set. It should be common practice to relay this information when reporting on the outcomes of each approach, as well as how each feature is associated with complications and symptom outcomes. In a subsequent study, it would be interesting to investigate which side of approach should be selected if the lesions are not lateralized/located near the center of the anterior cranial fossa.

Strategy of approach: The most commonly used surgical technique involves approaching the tumor from the non-dominant hemisphere in order to avoid complications related to the dominant hemisphere. However, we analyze the distribution of perilesional edema to determine the side of approach, taking into consideration the vascular supply and drainage from the lesion. Asymmetry of perilesional edema in the two hemispheres is an indication of a different displacement of the arachnoidal layers unilaterally, creating a better cleavage plane that protects the parenchyma. Variations in venous drainage are also of great relevance in protecting parenchymal function. It has become valuable practice in some centers to approach the tumor from the side with major edema, where this plane can be lost.

Approach: We also recommend that all studies involving open approaches clearly group patients into unilateral vs. bilateral categories. Furthermore, it is suggested to further subgroup them into anterior approaches (bifrontal, subfrontal, interhemispheric, frontomedial (eyebrow), or variations thereof) and lateral approaches (pterional, frontotemporal/supraorbital, or similar).

EOR: The extent of resection should be assessed radiographically at predefined intervals perioperatively (e.g., within 36 h, at three, six, and twelve months, and then annually) using predefined CT and MRI sequences that are agreed upon by the respective professional societies. For example, to accomplish consistent imaging, we recommend obtaining not only the standard T1/T2 pre- and post-operative MR images but also including pre- and postoperative FLAIR sequences, DWI, and MPRAGE sequences, as well as thin cut CT images in a bone window setting. This will allow all cases to determine lesional characteristics, the extent of pre and post-operative edema, infiltration of adjacent structures and bone, as well as any impact (stroke and swelling) on the frontal lobes due to the chosen approach. Bony infiltration should also be reported separately, as many centers have now adopted a more cautious approach in resecting these lesions in the elderly. They prefer to treat any residual disease or early recurrence with adjuvant radiation therapy or SRS.

Tumor histology: Tumor histology should be reported coherently and in accordance with the most recent literature (such as WHO grading and methylation pattern). Functional status needs to be regularly assessed at the same time points during the follow-up period. This methodology will provide a solid data base in the future to develop a standardized scheme that will aid in deciding which tumors are better suited for a particular treatment (i.e. stereotactic radiosurgery vs. surgery) or which surgical approach is likely to be most favorable.

**Figure 3:**
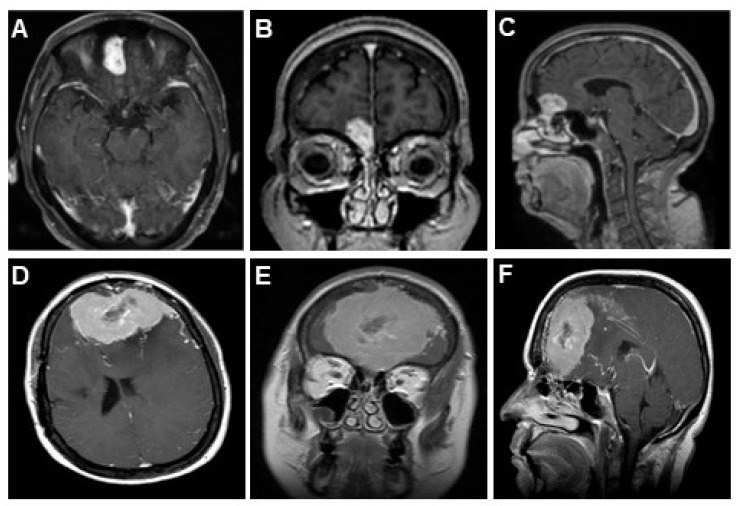
Pre-operative imaging of two Cases of OGMs. (**A**,**D**) Axial T1 gad, (**B**,**E**) coronal T1 gad, and (**C**,**F**) sagittal T1 gad of distinct cases of OGMs. Case 1 (**A**–**C**) and Case 2 (**D**–**F**).

Such a clear and streamlined scheme should allow the surgeon to assess which treatment modality would achieve the best EOR outcomes at the lowest possible morbidity, which will directly translate into better QOL outcomes for their patients. To demonstrate our approach, we show two representative case studies of patients with olfactory groove meningiomas (Figure 3). The first case is that of a medium OGM (Figure 3A–C, size < 3 cm, diameter = 2 points) without extensive edema, encasement of the ACAs, or dural infiltration, with unilateral extension (assign letter ‘U’), thus garnering a KMC score of 2-U. The second case is that of a massive OGM (Figure 3D–F, size > 5 cm, diameter = 4 points) with severe edema (added 1 point), ACA encasement (added 1 point), dural infiltration beyond the attachment point (added 1 point), and bilateral extension (assigned letter ‘B’), with a final KMC score of 6-B. We are currently performing an international study to validate patient outcomes when choice of surgical approach is guided by our OGM scoring system.

## 6. Conclusions

In our pooled analysis, it appears that unilateral approaches are superior to bilateral and straight frontal/subcranial approaches in minimizing iatrogenic morbidity while still achieving high rates of gross total resections. However, given the significant heterogeneity in reporting of currently available data, it is difficult to derive more specific conclusions regarding the optimal approach. It is also likely that the bilateral approach is chosen for very large tumors (Figure 3D–F), which introduces a potential bias to conclude that bilateral approaches carry a higher surgical risk. Therefore, we propose to validate our classification system in a future prospective study to address this bias. Our proposed future validation study will also help address the current limitations in assessing risk of bias and conducting statistical outcomes analyses based on surgical approach, which we are unable to perform in this current pooled analysis study.

In our practice, we think in terms of “bilateral vs. unilateral craniotomy”. The unilateral approach includes all craniotomies involving a lateral subfrontal corridor, whether through a pure frontal craniotomy or a basal fronto-temporal craniotomy. This approach appears to result in the lowest morbidity rates and improved QOL postoperatively. Although the bilateral approach may carry some advantages in terms of exposure and EOR, especially in very large tumors with a very anterior implantation base close to the crista galli, it also carries risks of increased morbidity due to frontal lobe retraction and potential development of brain edema and venous infarction. These factors need to be taken into account when dealing with frail patients. It must be emphasized that surgical approaches should ideally be tailored to the specific conditions that are found in each patient. Each approach may carry a distinct advantage based on the unique tumor morphology and configuration in a given patient. However, in the light of our review findings, it is clear that no stringent recommendation can be made regarding the preferred particular surgical technique, as there remains a gap in the literature correlating anatomical and radiographic features to specific outcomes. To this end, a comprehensive, rational, and reproducible classification system is required to generate meaningful data for comparing outcomes. Based on our observations, we propose an upgraded classification system for these tumors, which goes beyond size-based classifications. By adding several other descriptive variables of these lesions (such as extent of edema, extent and pattern of dural and osseous infiltration, vascular relationships etc.), we aim to encourage neurosurgeons to use this common language to create a standardized system in reporting symptoms and outcomes for these often-challenging tumors. We are currently conducting another study to review our own operative experience of OGMs, with the intention of presenting and validating our proposed classification scheme. These efforts are undertaken in order to find a concrete answer regarding the optimal approach for this relatively infrequent yet formidable anterior skull base pathology.

## Figures and Tables

**Table 1:** Surgical studies identified through our PRISMA analysis.

	Study	No. of Pts.	Tumor Size	Pterional	Unilateral Subfrontal	Bilateral Subfrontal	Supraorbital/ Frontolateral	Interhemispheric	EOR
1	Schaller (1994) [15]	28	3.5–6 cm	28					GTR: 27 STR: 1
2	Mayfrank (1996) [16]	18	1.5–7 cm					18 Unilateral Frontal Interhemispheric	GTR: 18
3	D’Avella (1999) [17]	6	6.5–9 cm	6					GTR: 6
4	Paterniti (1999) [18]	20		20					GTR: 20
5	Tsikoudas (1999) [1]	13	3–6 cm		2	11			GTR: 13
6	Turazzi (1999) [19]	37	4–6 cm	37					GTR: 37
7	Welge (2001) [20]	12	2–5.5 cm		8	4			GTR: 12
8	Hentschel (2003) [21]	13	3.5–8 cm			13			GTR: 11 STR: 2
9	Obeid (2003) [22]	15 9: DeNovo 6: Recurrent				15			GTR: 14 STR: 1
10	Spektor (2005) [23]	81		18	9	47 (12/47 Subcranial)	7		
11	Bassiouni (2007) [24]	56		13	4	36	3		GTR: 56
12	Colli (2007) [25]	17				17			GTR: 17
13	Nakamura (2007) [26]	82	1.4–10 cm	2		46 STR 3	34 STR 3		GTR: 76 STR: 6
14	Gazzeri (2008) [27]	36	5.6–8 cm	1		35			GTR: 31 STR: 5
15	Aguiar (2009) [28]	21	3.6–5.4	11		7	3		GTR: 13 STR: 8
16	El-Bahy (2009) [29]	18	<4 cm (7) >4 cm (11)				18		GTR: 14 STR: 4
17	Romani (2009) [9]	66	<3–>6				66		GTR: 60 STR: 6
18	Tomasello (2011) [30]	18		18					GTR: 17 STR: 1
19	Pepper (2011) [31]	19	2–7			19 (subcranial)			GTR:12 STR:7
20	Ciurea (2012) [32]	61	2–>6 cm	9	13	39			GTR: 53 STR: 8
21	Jang (2013) [33]	40				19	21		GTR: 37 STR: 3
22	Bitter (2013) [34]	61		61					GTR: 60 STR: 1
23	Ashish (2015) [35]	24	3–6 cm		5	19			GTR: 15 STR: 9
24	Pallini (2015) [36]	113	<3–>6 cm <3 (15) 3–6 (33) >6 (51)	21		92 (22/92 orbitofrontobasal)			GTR: 95 STR: 18
25	Guduk (2016) [37]	63	1.5–9 cm <3 (11) 3–6 (30) >6 (20)	38	25				GTR: 59 STR: 4
26	Nanda (2016) [38]	57		25		16	16		GTR: 52 STR: 5
27	Barzaghi (2017) [39]	21	2.5–7 cm			21 Trans-frontal sinus subcranial approach			GTR: 21
		1016		308 (30.3%)	66 (6.49%)	456 (74 subcranial variation) (44.88%)	168 (16.5%)	18 (1.77%)	GTR: 929 (91.4%) STR: 87 (8.6%)

**Table 2:** Post-operative complications at follow-up.

Study	Approach	Complications			Overall Mortality	Recurrence
		Minor	Major/Life Threatening	Non-Neurological		
Schaller (1994) [15]	Pterional		Hemorrhage (1)	Pulmonary Embolism & Death (1)	1/28 (3.6%)	
Mayfrank (1996) [16]	Unilateral Frontal Interhemispheric	Bone flap infection (1)	Persistent Coma, Ischemic infarct of the medulla (1)			
D’Avella (1999) [17]	Pterional	None				
Paterniti (1999) [18]	Pterional			Death from non-neurological issues (2)	1/20 (5%)	
Tsikoudas (1999) [1]	Bifrontal	Complete Anosmia (3) CSF Rhinorrhea (3) [Resolved spontaneously] Meningitis (1) Seizures (1) Blindness (1)		Pneumonia (1) MI (1)		4/13
Turazzi (1999) [19]	Pterional			Death from non-neurological issues (1)	1/37 (2.7%)	
Welge (2001) [20]	Bifrontal		Death from Malignant cerebral edema (1)		1/12 (8.3%)	
Hentschel (2003) [21]	Bifrontal	None				
Obeid [22] (2003)	Bifrontal	CSF Rhinorrhea (3) Worsening vision (1) CN Palsies (2) Seizure (1) Symptomatic Pneumocephalus (2)				
Spektor (2005) [23]						
Bassiouni (2007) [24]	Bifrontal Unifrontal Pterional Supraorbital	CSF Rhinorrhea (3) Worsening vision (1) CN Palsy (1) Seizures (1) (1.8%) one seizure preoperative, treated with Phenhydan Anterior pituitary insufficiency (1) None	Ischemic cerebral infarct from ACA injury (2) Venous infarction (1) Death (1) [Hemorrhagic infarction] Hemorrhage (1)	Death (2) [Pneumonia, Pulmonary embolism]	3/56 (5.4%)	5/36
Colli (2007) [25]	Bifrontal	Wound infection (4) Transient monoparesis (1) Seizures (2) Cerebral edema (1)	Death (1) [Hemorrhage]	Death (1) [Pneumonia]	2/17 (11.8%)	
Nakamura (2007) [26]	Bifrontal Frontolateral Pterional	Brain edema (7) Hydrocephalus (4) Subdural Hygroma (1) Seizure (2) CSF Leak (1) Infection (2) Subdural hygroma (6) Hydrocephalus (2) Cerebral Edema (1) Seizure (4) CSF Leak (2) Infection (1)	Hemorrhage (5) Deaths (3) [Malignant Cerebral Edema & Hemorrhage) Hemorrhage (1)	Death (1) [Pulmonary embolism]	4/46 (8.7%) Bifrontal group only	3/46 1/34
Gazzeri (2008) [27]	Bifrontal Pterional	CSF Leak (2) Seizures (1) Visual field deficit (1) Delayed pneumocephalus (1)		Death (1) [Pulmonary embolism]	1/36 (2.8%)	2/36
Aguiar (2009) [28]	Bifrontal Frontolateral	Hydrocephalus (4) CSF leak (5)	Death (1) [Cerebral Edema] Hemorrhage (1)		1/21 (4.8%)	4/21
El-Bahy (2009) [29]	Frontolateral	CSF Leak (3)	Death (1) [ACA injury, ischemic infarct] Hemorrhage (1)		1/18 (5.5%)	
Romani (2009) [9]	Supraorbital	CSF Leak (6) Hydrocephalus (2) Visual deficit (5) Wound infections (4)	Hemorrhage (1)			4/66
Tomasello (2011) [30]	Pterional	None				3/18
Pepper (2011) [31]	Bifrontal	CSF leak (3) Meningitis (1) Cerebral edema (3) Tension pneumocephalus (1)	Hemorrhage (2) Ischemic Infarct (1)			3/19
Ciurea (2012) [32]	Bifrontal Unifrontal Pterional	Seizures (29) Transient motor deficits (6) CSF leak (8)				7/61
Jang (2013) [33]	Frontolateral Bifrontal	CSF leak (2)	Hemorrhage (2)			3/40
Bitter (2013) [34]	Pterional	Seizures (5) CSF leak (2) Hydrocephalus (1) SDH/Seroma (5)		Pneumonia (1) Death (1) [Pulmonary Embolism]	1/61 (1.6%)	3/61
Ashish (2015) [35]	Bifrontal Unifrontal	CSF rhinorrhea (4) CSF collection (6) Meningitis (2)				
Pallini (2015) [36]	Bifrontal Orbitobasalfrontal Pterional	Meningitis (1) Wound infection (2) Seizures (1) Cerebral Edema (12) Hydrocephalus (4) CSF Leak (4) Wound infection (1) Seizures (1) Cerebral Edema (4) Hydrocephalus (1) Wound infection (1) Seizures (1) Hydrocephalus (1)	Hemorrhage (9) Ischemic Infarct (1) Death (4) Hemorrhage (1) Hemorrhage (1) Ischemic Infarct (1)		4/113 (3.5%)	
Guduk (2016) [37]	Pterional Unifrontal	Brain contusion (2) CSF leak (1) Wound infection (1) Hydrocephalus (1)	Hemorrhage (2)	Ischemic Infarct (1) → Death	1/63 (1.6%)	2/63
Nanda (2016) [38]	Pterional Frontolateral Bifrontal	Hydrocephalus (3) Wound infection (1) Meningitis (1) CSF leak (2) Meningitis (1) Wound infection (2)				5/57
Barzaghi (2017) [39]	TFSSA		Hemorrhage infarction (1)			1/21
		**226 (22.2%)**	**48 (4.7%)**		**22 (2.2%)**	**50 (4.9%)**

**Table 3:** Studies describing both open and endoscopic approaches.

Study	Almeida [50]	Banu [40]	Mukherjee [51]
**Pts.**	**20**	**19**	**33**
**Open Approach**	Bifrontal (10)	Pure EES (6) Supraorbital eyebrow—microscopic with endoscopic assistance (7) Combined EES with bicoronal or eyebrow microscopic approach (6)	Bifrontal 15 Unifrontal 12
**EES**	10		6
**EOR**	Bifrontal: GTR (9) STR (1) EES: GTR (7) STR (3)	Pure EES GTR (4) STR (2) Supraorbital with endoscopic assist GTR (7) Combined GTR (6)	GTR: 28 STR: 5
**Pre-op** **symptoms**		Headaches (4) Olfactory (6) Improved (0/6) Cognitive (7) Seizures (2) Visual (4) Improved (4)	
**Post-operative symptoms**			
**Recurrence**	2/20 (1 in each group)	3 (2 in EES, 1 in combined)	2/33
**Complications**	Bifrontal: CSF Leak (1) Meningitis (1) EES CSF Leak (1) Meningitis (1) Abscess (1)	EES: CSF leak/pneumocephalus (1) Anosmia (6) Hemorrhage (2) Infection (2) Supraorbital Anosmia (4) Combined: Visual (1) Anosmia (6) Infection (1)	CSF Leak (7) [LD, 2 EES fix] Cerebral Edema (3) [Decompression] → Death in (1) Seizures (2) Wound Infection (2) Hydrocephalus (1) Hemorrhage (2) [Required Evacuation]

## Data Availability

Data is available upon request.
